# Fevers

**Published:** 1904-08-20

**Authors:** 


### FEVERS.

(*Continued from page 329.*)

Typhoid Fever (*continued*).—The occurrence of rigors in typhoid fever apart from recognisable complication is dealt with by C. Bolton.[8] He describes a remarkable case which occurred in University College Hospital under the care of Dr. Martin. The patient, a nurse, aged 24, was discharged cured on the one hundred and fifteenth day of the disease. During this long illness, lasting four months, 21 rigors occurred. The case presented the ordinary features of a severe attack. Diarrhœa and hæmorrhage were present, but ceased on the twenty-third day. Beyond a very trivial and transitory swelling of the legs, considered to be of toxic or hydræmic origin and not to be due to thrombosis, no complication was found. The rigors occurred in groups. The earlier ones, some of which were coincident with an attack of diarrhœa, were extremely severe. On three occasions the temperature exceeded 107° F., the highest recorded point being 107·4° F. on the twenty-fifth day of illness. The pulse during the severe rigors was markedly affected, being rapid, weak, and irregular. On one or two occasions it was uncountable, and the patient was apparently only saved from death by saline infusions and strychnine. Very little alcohol was used throughout. The blood examination for malarial parasites was negative, and quinine had no influence on the rigors. On reviewing the literature on the subject Bolton finds a good many similar, if less severe, cases have been recorded by Abercrombie, Bouveret, Bryant, Church, Frenkel, Herringham, Poole, and others. Mental shock, constipation, intestinal irritation, treatment by antipyretics, or external applications are among the possible causes suggested by these writers. Other cases are ascribed to thrombosis of mesenteric or small internal veins, small abscesses in liver or kidneys, malaria, or septic absorption. Irritation of the intestinal mucous membrane is considered to best explain the present case. Many of the cases seem to recover, and the prognosis is not materially affected by the presence of this phenomenon. The occurrence of hitherto unsuspected *meningitis* in many infectious diseases has been revealed by the modern method of lumbar puncture. The same procedure has also shown that recovery is possible in meningitis, even in the suppurative form. M.M. Achard and Paisseau[9] have recently described a case of typhoid fever accompanied by meningitis. The patient, a lad of 18 years, suffered from headache, repeated rigors, vomiting, constipation, and rigidity of the neck. Kernig's sign was well marked. Symptoms of typhoid were also present, and Widal's reaction was obtained later. On lumbar puncture clear fluid with abundant lymphocytes was obtained. Marked relief of the meningeal symptoms followed the puncture. The fluid on cultural examination was found to be sterile, showing that the meningitis was due to the action of toxins. M. Netter[10] has found meningitis associated with typhoid fever in 44 out of 313 cases, and also with pneumonia. He regards Kernig's sign as a most reliable indication of spinal irritation. D. Scannell[11] has described a case of myositis of the rectus abdominis following an attack of enteric fever complicated by the presence of boils and an ischiorectal abscess. The patient was a boy aged nine. The myositis, which subsided, simulated acute perforative appendicitis, but on operation the appendix was found normal. Attention has been directed to the occurrence of *desquamation*[12] in typhoid fever. Generally the face, palms, and soles escape. W. S. Thayer[13] has made a study of the after-history of typhoid cases as regards their cardiac and vascular condition. One hundred and eighty-three patients who had been treated for typhoid in Professor Osler's wards were examined. In these cases the average blood-pressure was found raised, in many cases it exceeded the healthy normal limit. The radial arteries were found palpable in old typhoid cases three times as often as in individuals who had not had the disease. Increase of blood-pressure and hypertrophy of the heart were most marked in those cases who during their attack had exhibited irregularity and rapidity of pulse, and the onset of murmurs. Dr. Thayer concludes that the typhoid poison is an important factor in inducing arterio-sclerotic changes and cardiac hypertrophy. There would seem to be grounds for the belief that next to rheumatic fever typhoid exercises of all infectious maladies the most harmful influence upon the heart. The diagnosis of enteric is dealt with by Claude B. Ker,[14] who, while recognising the diagnostic difficulties often met with clinically, indicates the lines on which it should be attempted. The onset is generally insidious, although occasionally cases start abruptly. If a patient complains for a week or 10 days of *malaise*, anorexia, loss of sleep, chilliness, headache, and the vague bodily pains which accompany a raised temperature from any cause, together with symptoms pointing to disturbance of the alimentary canal, such as abdominal pain, diarrhœa, or, a less valuable sign, constipation, and if, at the time of examination, fever is present, that patient is probably suffering from enteric. A history of slight cough, and especially of epistaxis, increases the probability. Wide dilatation of pupils is common in the first fortnight. During this time, also, a dicrotic pulse, slow in proportion to the temperature, is characteristic. Tumidity of the abdomen is the rule; flatness or hollowness, on the contrary, is against typhoid. Tenderness in the right iliac fossa is valuable. The presence of typical spots is final; their absence is of no value. In differential diagnosis careful examination of the chest, repeated for several days until physical signs develop, will help to distinguish enteric fever from acute lobar pneumonia—a common source of error in hospital cases. Labial herpes common in pneumonia is rare in enteric. Tubercular conditions are sometimes extremely difficult to differentiate: acute miliary tuberculosis is especially so. Decided lividity of the face and extremities, often present in these cases, is the most useful indication to go upon. Sometimes Widal's reaction alone makes a diagnosis possible. Decided symptoms of tubercular meningitis are sometimes present. Rarely the two diseases may coexist. A very slow pulse in proportion to the temperature favours meningitis. Choroidal tubercles or typhoid spots, when they are present, are decisive. Influenza presents a difficulty at first, but the course is shorter. Marked frontal headache and coryza favour influenza. The diazo-reaction in enteric is chiefly valuable as a negative sign. It occurs also in acute tuberculosis, pneumonia, and the eruptive fevers. Isolation of the bacillus typhosus from the blood, spots, or stools is of course conclusive, but a negative examination is valueless. Ker regards the Widal test, properly carried out, after the second week, as practically infallible. A previous attack and paratyphoid infections are the only sources of error.

[8] Pract., Jan. [9] Lancet, May 14, p. 1368. [10] Ibid. [11] Ibid., Feb. 27, p. 593. [12] Amer. Jour. Med. Sc., Jan., p. 57. [13] Med. Rec., April 2, p. 537. [14] Pract., March.

(*To be continued.*)

